# Personalised Interventions for Self-Harm in High-Risk Adolescents: A Case Report

**DOI:** 10.7759/cureus.109188

**Published:** 2026-05-19

**Authors:** Sabīne Bebere, Zane Semjonova, Liene Vītola

**Affiliations:** 1 Department of Psychiatry, Children's Clinical University Hospital, Riga, LVA; 2 Department of Residency, Rīga Stradiņš University, Riga, LVA

**Keywords:** adolescent psychiatry, case report, emotional dysregulation, self-harm, self-harm reduction

## Abstract

Self-harm in adolescents is a complex clinical challenge often driven by profound emotional dysregulation and complicated by trauma history. This report describes the case of a 14-year-old female patient with a history of sexual abuse, post-traumatic stress disorder, and major depressive disorder, who was repeatedly hospitalised for severe self-inflicted wounds. Despite multiple hospitalisations following suicide attempts and low initial motivation for psychotherapy, a multidisciplinary team implemented a modified therapeutic approach. Due to the patient’s inability to participate in high-intensity dialectical behaviour therapy (DBT), a customised harm-reduction strategy was employed, featuring formal behavioural agreements, environmental controls, and a tangible reward system. The core component of this approach was a structured behavioural agreement, which was formulated by the clinical team, the patient, and her mother, to maintain environmental control and promote transparency. This intervention focused on stabilising wound severity and improving transparency rather than immediate cessation. Over the course of treatment, this individualised strategy led to a significant reduction in self-harm frequency, improved treatment adherence, and served as a clinical bridge towards more intensive therapeutic engagement. The case suggests that for high-risk adolescents with fluctuating motivation and treatment nonconformity, customised harm-reduction and modified DBT-informed strategies can effectively manage immediate risk while fostering long-term recovery.

## Introduction

Self-harm, such as intentional self-poisoning or injury, regardless of the apparent purpose of such action, is a growing problem in most countries, often repeated, and associated with suicide [1,2]. Methods that have a high chance of being fatal are more likely to be associated with serious psychiatric disorders. Self-harm is more commonly seen in women than in men and in young people than in older adults [3]. The term non-suicidal self-injury (NSSI) or non-suicidal self-harm describes intentional direct damage to one's own body tissue without the intention of ending one’s life, for example, by cutting or burning the skin. There is inconsistent evidence about whether NSSI and suicidal behaviour represent separate diagnostic entities or whether they fall along a single continuum of self-harm. Nonetheless, there is adequate evidence indicating that NSSI increases the risk of later suicide attempts. In some instances, NSSI can have potentially life-threatening outcomes even when suicide is not the intended goal, making it essential to evaluate both the lethality of the behaviour and the person’s intent while performing a safety assessment [4]. Most self-harm patients have anxiety and depressive symptoms in a backdrop of chronic social and personal difficulties, often complicated by substance use. They usually do not require psychotropic medication or specialised psychiatric treatment but may benefit from individual psychological support [3]. Adolescents who engage in self-harm have greater difficulties in identifying, describing, understanding, and expressing their own feelings, leaving them at risk of turning to maladaptive means as ways to regulate difficult emotions. The self-mutilation act can be a specific method of coping with emotional distress for individuals who lack more adaptive coping strategies [5]. Although many adolescents benefit from psychological interventions, participation in treatment can be inconsistent, particularly in those with complex psychosocial backgrounds. In this paper, we report the case of a high-risk adolescent with trauma history and severe emotional dysregulation, hospitalised for repeated self-harm with a sharp object, for whom standard high-intensity interventions were initially unfeasible because of her lack of motivation. The case illustrates managing severe emotional dysregulation when abstinence-only goals are unattainable, requiring a modified approach based on a structured behavioural agreement and active maternal involvement to ensure environmental safety and clinical transparency.

## Case presentation

History and presenting concerns

A 14-year-old female presented to the outpatient psychiatric clinic (Month 1) with a four-year history of self-harm, progressing from superficial scratching to deep lacerations. Previously, the patient had been hospitalised at another state psychiatric hospital, where she was prescribed sertraline, haloperidol, and valproic acid as part of her treatment. The patient’s history is notable for childhood sexual abuse perpetrated by a stepfather when she was eight years old, resulting in ongoing legal proceedings and the removal of the stepfather from the home. Despite the removal, the patient reported significant guilt and ambivalence about reporting him. It is also known that the stepfather was abusing alcohol. Additional stressors included chronic school bullying and the recent ending of a close friendship. Comorbid symptoms included school avoidance, nausea, headaches, reduced appetite, and sleep inversion from nocturnal gaming. (See full timeline in Table 1.) The initial diagnoses following the first outpatient consultation were major depressive disorder, social phobia and deliberate self-harm using a sharp object.

**Table 1:** Timeline of Clinical Events and Interventions. DBT, dialectical behaviour therapy.

Time period	Clinical status and key events	Interventions
Month 1	Suicidal ideation; overdose on sertraline, carbamazepine, and haloperidol.	Admission to ICU; legal proceedings continued against stepfather. Initiated treatment with venlafaxine and olanzapine.
Month 3	Recurrence of suicidal thoughts.	Two-week psychiatric hospitalisation followed by a day-care program. In therapy, add oxcarbazepine.
Month 9	Severe self-harm (1 by 8 cm wounds); patient is uncritical of actions.	Emergency admission to the psychiatry ward; development of a new safety plan.
Month 9.5	Transition to outpatient care with fluctuating motivation.	Implementation of harm-reduction agreement and medication (venlafaxine, olanzapine, oxcarbazepine).
Month 10	Violation of agreement; severe wound to breast (3 by 5 cm).	Brief re-hospitalisation as a clinical consequence.
Month 11-12	Improved adherence to DBT journal; abstinence from significant self-harm.	Weekly positive reinforcement (stickers).
Month 14	Clinical stability is maintained in the day-care program.	Continued monitoring and eligibility for larger tangible rewards.

Clinical findings and examination (Month 9)

During the initial emergency hospitalisation in Month 9, the patient was alert and oriented; she exhibited a significant lack of insight regarding her condition, dismissively downplaying the severity of the lacerations on her upper arms. The mental status examination (MSE) revealed a withdrawn, depressed mood. A defining feature of her presentation was the ego-syntonic nature of her self-destructive impulses, which rendered her defenceless against the urge to self-harm. The frequency of self-harm episodes appears to be closely linked to internal affective triggers rather than external stressors alone, indicating a primary deficit in emotional regulation. A detailed physical examination documented a high density of NSSI markers. Physical examination revealed between two and 23 small healing wounds on each thigh and two large, secondary-healing lacerations on the upper arms. Among these was a freshly inflicted wound on the left upper arm, measuring 2 by 10 cm, characterised by gaping margins and involving the subcutaneous layer, which necessitated immediate wound care. The presence of both chronic and acute injuries underscored a pattern of escalating self-injurious behaviour and an immediate need for clinical stabilisation. Specifically, a shift from superficial cuts to deep-tissue lacerations suggests a progressive desensitisation to pain and an increasing threshold for emotional relief. 

Therapeutic intervention 

The patient was stabilised on a pharmacological regimen of venlafaxine (150 mg/day), olanzapine (2.5 mg/day), and oxcarbazepine (300 mg/day). The addition of Oxcarbazepine during Month 3 was specifically indicated to address her marked behavioural volatility and impaired impulse control, providing a mood-stabilising effect. Following a history of overdose with sertraline and haloperidol, these agents were strictly contraindicated, and oxcarbazepine was utilised as a clinically appropriate alternative to address behavioural dysregulation while avoiding pharmacological agents associated with the patient’s prior suicide attempts. A multidisciplinary team (psychologist, occupational therapist, and psychiatrist) focused on a safety plan. Given her resistance to high-intensity psychotherapy, a Harm-Reduction Agreement was established to provide a behavioural framework; this strategy actively integrated the patient's mother to ensure consistent environmental control and monitoring at home (see Table 2). Outpatient compliance was monitored through weekly clinical body examinations to provide objective verification of wound parameters, bypassing her unreliable self-reporting and evident pride in her injuries. This was supplemented by maternal oversight to ensure environmental safety and the immediate disclosure of new wounds. Ultimately, risk assessment was continuously calibrated based on the patient’s adherence to these transparency requirements and her engagement with a dialectical behaviour therapy (DBT) journal, balancing her poor insight with a pragmatic approach to long-term safety.

**Table 2:** Structured Harm-Reduction and Behavioural Reinforcement Agreement.

Domain	Intervention, rule
Wound parameters	Agreement to limit self-harm to the epidermis; wounds must not exceed 5 cm in length or 2 mm in width.
Transparency	Mandatory immediate disclosure of any new wounds to the mother to ensure proper hygiene and infection control.
Environmental control	Restriction of unsupervised online shopping and automatic payments; minimal access to sharp objects at home.
Clinical monitoring	Mandatory weekly outpatient body examinations every Thursday at 10:30 a.m.
Positive reinforcement	Weekly reward (sticker) for compliance; a larger tangible reward (platypus toy) earned after three months of adherence.
Consequences	Immediate re-hospitalisation if the agreement is violated.

Outcomes and follow-up 

The patient was discharged with the following diagnoses: major depressive disorder, post-traumatic stress disorder, and deliberate self-inflicted injury using a sharp object. Following a brief re-hospitalisation in Month 10 due to a significant agreement violation, the patient showed improved engagement with DBT journaling. The patient exhibited a progressive decline in both the frequency and clinical severity of self-harming behaviours. By the 12-month mark, the patient had achieved clinical stabilisation, characterised by cessation of significant self-harming behaviour and absence of newly reported tissue injuries. At Month 14, the patient remained clinically stable and sustained her abstinence, thereby fulfilling the requirements for the tangible reward she had selected.

## Discussion

This case highlights the issues and challenges clinicians can face with self-harming patients. The medical history showed that the patient had difficulty complying with the agreement on self-harm; for a long period of time, she continued making deep cuts and not seeking treatment. Adolescents can be exposed to stressful life situations that can strongly influence their mental health, and for which they usually do not yet have the necessary coping skills or resources to deal with [6]. In the present case, the patient had been exposed to a traumatic event, sexual abuse, which was associated with a subsequent deterioration in her capacity for emotional regulation. Current evidence suggests that reducing NSSI in adolescents requires a multimodal approach that combines evidence-based psychotherapy, targeted emotion-regulation interventions, and system-level support [7]. In the presented case, treatment involved some DBT methods, as the full DBT was too intense for the patient. DBT does not allow avoidance of painful emotions; instead, it requires engaging with them to build tolerance, which can be distressing. Nevertheless, these elements were introduced to support the development of alternative coping strategies and to gradually strengthen treatment engagement. At the beginning of accepting psychological support, she admitted that she might not be motivated and often refused to reflect more deeply about her feelings and experiences. At a day-care centre, she visited a psychologist who guided her progress and received physiotherapy and occupational therapy. The patient started using a DBT diary but was not enrolled in the full programme. Self-harm behaviours persisted, indicating the need for additional motivational low-intensity interventions. Therefore, a harm-reduction strategy was implemented, including an agreement to limit the depth of self-inflicted cuts, ensure appropriate wound care, and introduce positive reinforcement during periods without significant self-harm, such as using stickers and toys. The ethical foundation of this strategy rested on a shift from an 'abstinence-only' model that had previously failed this patient to a pragmatic harm-reduction framework. By collaboratively defining 'safe' boundaries with the patient and her mother, the clinical team established a transparent monitoring system. This prevented the normalisation of self-harm by maintaining a strict 're-hospitalisation' consequence for violations. At the same time, the age-appropriate reward system provided positive reinforcement for the patient’s willingness to remain within a medically manageable safety range during her transition to more intensive interventions. As motivation grows, DBT remains a viable option for the future, as it is clear that this method would be useful. Reducing repeated hospital admissions represented an important clinical objective in this case, as prolonged or recurrent psychiatric hospitalisation can unintentionally reinforce self-harm behaviours in vulnerable adolescents. Given the patient’s history of multiple admissions and ongoing emotional dysregulation, emphasis was placed on maintaining treatment within outpatient and day-care settings whenever clinically feasible. Alternatives to inpatient care, including intensive community-based support and structured follow-up, can therefore provide a more adaptive treatment framework. More recently, internet-based interventions such as Internet-Based Emotion Regulation Therapy for Adolescents (IERITA) have emerged as promising and tangible options to reduce self-injurious episodes [8]. Although qualitative work suggests that these are acceptable to youth, heterogeneity in study designs limits broad applicability [6]. While these interventions remain vital considerations for other clinical cohorts, they were less applicable in the presented case due to the patient's acute trauma history and treatment nonconformity. For high-risk cases, a multimodal approach that combines established therapy with active family participation remains the empirically supported strategy [7]. Since 2025, a pilot project has been underway in Latvia to adapt and implement an evidence-based model of semi-automated online cognitive behavioural therapy (iCBT) for the treatment of mild depression, generalised anxiety, and social anxiety among Latvian adolescents and young adults. This approach represents a form of internet-integrated therapy designed for the treatment of less severe mental health conditions [9]. Consequently, it is not intended for individuals who present with severe self-harm or high clinical risk. In the present case, online interventions could not be applied because the girl’s psychological condition was too severe to meet the clinical indications for iCBT. In general, the literature supports a flexible, individualised treatment strategy in which established therapies, such as CBT and DBT-A, form the clinical backbone, while tangible interventions focusing on emotion regulation, such as IERITA, are integrated when appropriate. A challenge is to design and evaluate scalable, brief interventions that non-specialist or low-intensity practitioners can deliver to meet the needs of young people who may not be offered high-intensity interventions such as DBT. Although there is some evidence of a lower rate of self-harm repetition for children and adolescents receiving DBT-A compared to those in a control group after intervention, most other interventions developed to date, regardless of their specific therapeutic approach, are associated with similar effects for repetition of self-harm as with comparator treatments (e.g., cognitive behavioural-based therapy; mentalisation-based therapy for adolescents). This suggests that current treatment approaches may not adequately address the underlying psychological processes that drive self-harm behaviour [10].

## Conclusions

This case illustrates the clinical complexity of managing adolescent self-harm, particularly in the context of limited treatment adherence, fluctuating motivation, and ongoing emotional dysregulation. Behavioural agreements and harm-reduction strategies, although not curative, served as pragmatic interim measures to reduce immediate risk. This case report highlights the value of offering individually tailored interventions, for example, by first applying selected DBT strategies and a reward system before moving on to more intensive methods (full DBT). In general, the report underscores the importance of continuously developing and assessing adaptable treatment approaches that address key drivers of self-harm, especially emotional dysregulation, while balancing clinical efficacy, patient participation, and practical service delivery.
